# Femoral Neck Design Does Not Impact Revision Risk After Primary Total Hip Arthroplasty Using a Dual Mobility Cup

**DOI:** 10.1016/j.artd.2023.101281

**Published:** 2024-01-13

**Authors:** Bart van Dooren, Rinne M. Peters, David Visser, Liza N. van Steenbergen, P Koen Bos, Wierd P. Zijlstra

**Affiliations:** a Department of Orthopaedic Surgery, University Medical Center Groningen, Groningen, The Netherlands; b Department of Orthopaedic Surgery, Medical Center Leeuwarden, Leeuwarden, The Netherlands; c Department of Orthopedic surgery, Martini Hospital, Groningen, The Netherlands; d Department of Orthopedics and Sports Medicine, Erasmus Medical Center, Rotterdam, The Netherlands; e Dutch Arthroplasty Register (LROI), ‘s-Hertogenbosch, The Netherlands

**Keywords:** Dual mobility, Wear, Intraprosthetic dislocation, Neck geometry

## Abstract

**Background:**

The use of dual mobility (DM) cups has increased quickly. It is hypothesized that femoral neck taper geometry may be involved in the risk of prosthetic impingement and DM cup revision. We aim to (1) explore the reasons for revision of DM cups or head/liners and (2) explore whether certain femoral neck characteristics are associated with a higher risk of revision of DM cups.

**Methods:**

Primary total hip arthroplasties with a DM cup registered in the Dutch Arthroplasty Register between 2007 and 2021 were identified (n = 7603). Competing risk survival analyses were performed, with acetabular component and head/liner revision as the primary endpoint. Reasons for revision were categorized in cup-/liner-related revisions (dislocation, liner wear, acetabular loosening). Femoral neck characteristics were studied to assess whether there is an association between femoral neck design and the risk of DM cup/liner revision. Multivariable Cox proportional hazard analyses were performed.

**Results:**

The 5- and 10-year crude cumulative incidence of DM cup or head/liner revision for dislocation, wear, and acetabular loosening was 0.5% (CI 0.4-0.8) and 1.9% (CI 1.3-2.8), respectively. After adjusting for confounders, we found no association between the examined femoral neck characteristics (alloy used, neck geometry, CCD angle, and surface roughness) and the risk for revision for dislocation, wear, and acetabular loosening.

**Conclusions:**

The risk of DM cup or head/liner revision for dislocation, wear, and acetabular loosening was low. We found no evidence that there is an association between femoral neck design and the risk of cup or head/liner revision.

## Introduction

Recurrent dislocation is the most common cause of early revision in total hip arthroplasty (THA) [1]. Both patient-, surgeon-, and procedure-related factors have been associated with THA dislocation [[2], [3], [4], [5]]. Dual mobility (DM) cups have been developed to reduce dislocation rates, and their use has increased quickly [5]. Unlike unipolar cups, DM cups carry a different failure scenario. DM cups commonly fail due to liner wear, acetabular loosening, intraprosthetic dislocation (IPD), and prosthetic impingement, while unipolar cups frequently fail due to loosening and dislocation [[6], [7], [8], [9], [10], [11]]. Based on clinical experience and expert opinion from France, it is assumed that neck taper geometry may be involved in the risk of DM cup revision [9,12]. Unlike fixed bearings, the mobile polyethylene (PE) insert in DM cups can freely rotate in its metallic shell, which increases joint mobility. However, extreme movements may cause femoral-neck impingement. It is hypothesized that neck geometry, particularly increased thickness, may lead to contact and impingement with the PE liner, reducing clearance, leading to more friction and PE wear, ultimately compromising the liner's retentive power and raising the risk of IPD. The obstructed movement of the outer articulation and unexpected liner-neck impingement can subsequently contribute to wear-related problems and, in more severe cases, even lead to the risk of acetabular loosening. Moreover, surface roughness and femoral neck irregularities, particularly in noncylindrical or rough surfaces, may potentially exacerbate wear by creating abrasive interactions with the PE material. Hence, smooth, polished, and narrow necks might reduce the risk of DM cup failure. Moreover, it is hypothesized that the prosthesis neck-to-shaft (CCD) angle impacts the range of motion of THA, which, in turn, affects the risk of impingement [13]. Except for these anecdotal reports, there is limited evidence to support this hypothesis. It is vital to better understand the modes of failure and to monitor which femoral neck characteristics may be less optimal in combination with a DM cup. Evaluation of various femoral neck design features and mechanism of failure using registry data can help to avoid unfavorable implant choice.

In this study, we aim to (1) explore the reasons for revision of DM cups and head/liners and (2) explore whether specific femoral neck design-related factors are associated with a higher risk of revision of the DM cup or head/liner in primary THA, specifically focusing on liner wear, dislocation, and acetabular loosening. We hypothesize that femoral neck design, including a thick neck, rough surface, and noncylindrical geometry, is associated with an elevated risk of revision for wear, dislocation, and acetabular loosening.

## Material and methods

### Study design

This study represents a retrospective population-based cohort study including all primary THAs with a DM cup in the Netherlands. Data from January 1, 2007, to December 31, 2020, were retrieved from the Dutch Arthroplasty Register (LROI), a nationwide population-based register. The database has a 98% completeness rate for registered primary THAs in the Netherlands since 2015 [14]. Ethical approval was not required according to the Dutch Medical Research Involving Human Subject Act.

**Figure 1:**
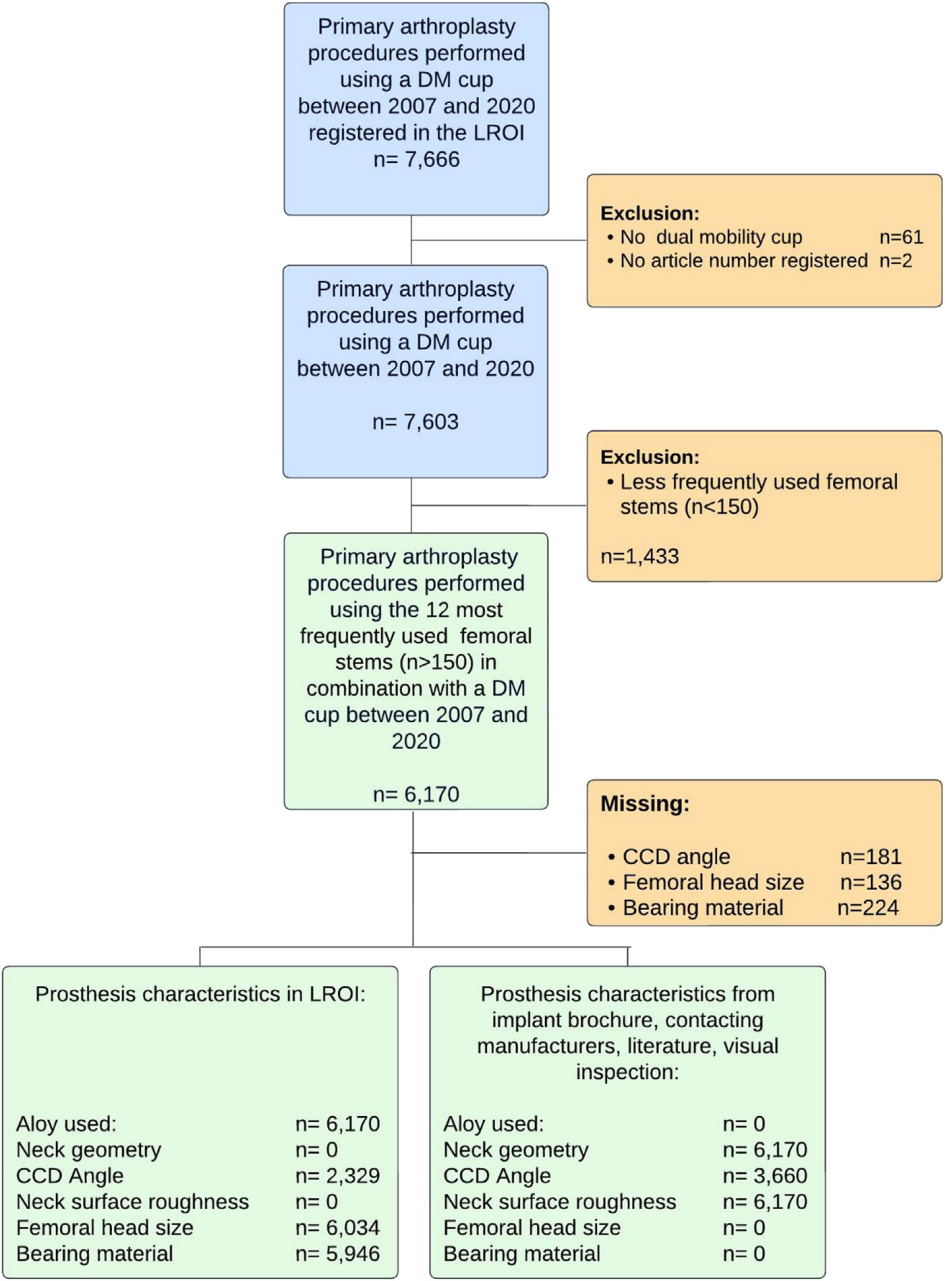
Flow chart of included procedures for analyses.

We included all primary THAs with a DM cup in the period 2007-2020 (n = 7603) (Fig. 1). Cups without a product code or those with a product code other than “dual mobility” were excluded (n = 63). Second, to determine femoral neck characteristics, we identified the 12 most frequently used (n > 150) femoral stems (n = 6170). Femoral components with less than 150 cases (n = 1433) were excluded (Fig. 1).

### Variables

**Figure 2:**
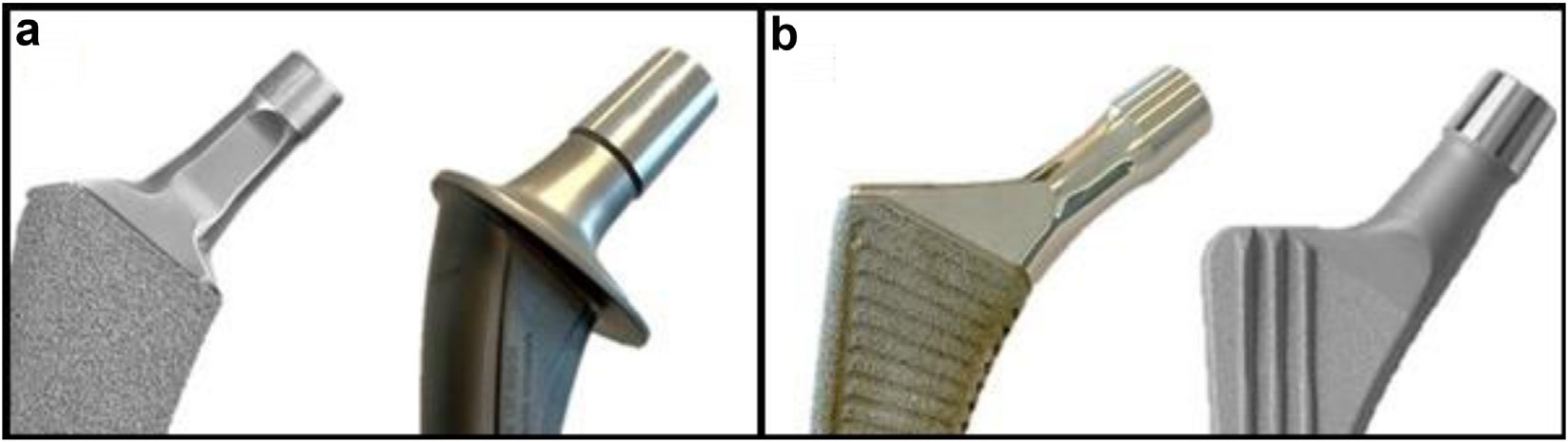
Examples of different femoral stems with varying neck designs; (a) trapezoidal neck design (Taperloc Complete) vs cylindrical neck design (Lubinus SPII); (b) highly polished surface polish (Polar Stem) vs matte surface polish (CLS Spotorno).

Demographic data, procedure, prosthesis characteristics, outcome measures, and data regarding stem and cup manufacturer as well as model names were provided. We collected the following patient demographics from the LROI: age, sex, body mass index (BMI), American Society of Anesthesiology (ASA) score, and preoperative diagnosis. In addition, information regarding prior surgeries and the chosen surgical approach was collected from the database. Moreover, implant-specific data for the most frequently used femoral components were collected. The following characteristics were retrieved from the LROI: femoral brand, model name, alloy used (titanium, cobalt chrome, and stainless steel), head size, bearing material, fixation methods, CCD angle, and articulation type. LROI did not provide information on 2 characteristics: Neck geometry (cylindrical or rectangular/oval) and surface roughness (polished/highly polished or matte/smooth). To obtain information on neck geometry and surface roughness, publicly accessible sources such as implant brochures, technical manuals, and relevant articles were used [[15], [16], [17], [18], [19], [20], [21], [22], [23], [24], [25], [26], [27], [28], [29], [30], [31]]. Moreover, information on design specifications was obtained by contacting different manufacturers. Finally, in cases where data were missing in implant brochures, the authors estimated the characteristics by visually examining prostheses and pictures (Fig. 2a and b). For every implant examined, any additional information regarding femoral neck characteristics was integrated into our existing data set derived from the LROI. For a comprehensive overview of our data sources and the extent of missing data, please refer to Figure 1 and Table 1.

**Table 1:** Overview of prosthesis characteristics/femoral neck design of the most frequently used femoral components in combination with a dual mobility cup in the Netherlands between 2007 and 2020.

Brand^a^	Type femur component^a^	n^a^	Alloy^a^	Taper^b^^,^^c^	Neck geometry^c^^,^^d^^,^^e^	CCD angle^a^^,^^b^	Neck surface^b^^,^^c^^,^^e^
Zimmer Biomet	Original ME Muller	1426	Cobalt chrome	12_14 [15]^b^	Cylindrical^e^	135 [15]^b^	Matte/Fine blasted^e^
Zimmer Biomet	STANMORE	701	Cobalt chrome	Type 1 taper [23]^b^	Cylindrical^e^	130 [23]^b^	Satin surface finish [23]^b^
Zimmer Biomet	Taperloc Complete	302	Titanium	12_1 [16,17]^b^	Rectangular/oval [30]^d^	133 [16,17]^b^	Polished [16,17]^b^
Zimmer Biomet	CLS Spotorno	250	Titanium	12_14 [18,29]^b^	Cylindrical^e^	125/135/145^a^	Matte/Grit blasted [18,29]^b^
Zimmer Biomet	Alloclassic Zweymuller	197	Titanium	12_14 [19]^b^	Cylindrical^e^	131^a^	Matte/Fine blasted [19]^b^
Zimmer Biomet	M/L Taper	174	Titanium	12_14 [20]^b^	Rectangular/oval [30]^d^	Unknown	Highly polished^e^
Zimmer Biomet	Wagner Cone	172	Titanium	12_14 [21,22]^b^	Rectangular/oval [30]^d^	125/135^a^	Matte/Corundum blasted [21,22]^b^
Smith & Nephew	Polar Stem	161	Titanium	12_14 [24]^b^	Cylindrical [30]^d^	126/135^a^	Highly polished^e^
Smith & Nephew	Spectron EF	175	Cobalt chrome	12_14 [25]^b^	Rectangular/oval^e^	131 [25]^b^	Polished^e^
Stryker	Exeter V40	762	Stainless steel	V40 Taper [26]^b^	Cylindrical^e^	125 [26]^b^	Highly polished^d^
DePuySynthes	Corail	294	Titanium	12_14 [27]^b^	Rectangular/oval^c^	135 [27]^b^	Highly Polished^c^
Link Lima	Lubinus SP2	1556	Cobalt chrome	12_14 [28]^c^	Cylindrical^c^	117/126/132^a^	Matte/smooth^c^

a Data obtained from LROI.

b Data obtained from implant brochure.

c Data obtained by contacting manufacturers.

d Data obtained through literature.

e Data obtained by visually examining prostheses.

### Outcome measures

First, we examined reasons for DM cup/liner revision. Reasons for revisions were categorized into cup-/liner-related revisions (dislocation, liner wear, and acetabular loosening) and other revisions (infection, periprosthetic fracture, and femoral loosening). Second, we examined the DM cup or head/liner revision rate for wear, dislocation, and acetabular loosening. Revision was defined as any change, addition, or removal of the cup, head, and/or liner. Crude cumulative incidence of DM cup or head/liner revision (for dislocation, wear, or acetabular loosening) was calculated.

### Femoral neck characteristics

Finally, the risk of DM cup/liner revision for wear, dislocation, and acetabular loosening was investigated for different femoral neck characteristics. Femoral stems with comparable femoral neck characteristics were pooled to examine if any specific femoral neck design features were associated with a higher risk of revision. The following femoral neck characteristics were analyzed: (1) alloy used; (2) femoral neck geometry; (3) CCD angle; (4) neck surface roughness; (5) femoral head size; and (6) bearing material. A detailed overview of included femoral stem and implant-related parameters for the most frequently used femoral components is given in Table 1.

### Statistics

Survival time was calculated as the time from primary DM THA to first revision arthroplasty, death of the patient, or the end of the follow-up period (January 1, 2021). The crude cumulative incidence of cup or head/liner revision for dislocation, liner wear, or acetabular loosening was calculated using competing risk analysis. After pooling specific femoral neck design features, we explored whether any specific femoral neck design features were associated with a higher risk of dislocation, wear, and acetabular loosening. Multivariable Cox proportional hazard regression analyses were performed to compare adjusted revision rates between different femoral neck characteristics. In addition, femoral head and liner characteristics were added to the model, including femoral head size (22 or 28 mm) and bearing material (standard PE, highly cross-linked polyethylene [HXPLE], or HXPLE + antioxidant). Adjustments were made for sex, age, ASA score, and diagnosis at primary procedure. BMI was not included as a covariate, since BMI was only available in the LROI database since 2014. The assumption of proportional hazards was verified by inspecting Schoenfeld residuals. Results were reported as hazard ratios (HR) with 95% confidence intervals (CI). Statistical analyses were performed using SPSS (Version 14.0) and R Statistical Software (version 2022.12.0: R foundation for Statistical Computing, Vienna, Austria). For all tests, a two-tailed significance level of *P* < .05 was used.

## Results

A total of 7603 primary THAs using a DM cup were registered in the LROI between 2007 and 2020. The median follow-up duration was 2.4 years (range 0-13). The median age (standard deviation) was 73 (12) years. Sixty-five percent of patients were female, 62% had ASA grade I-II, and 52% received THA for osteoarthritis (Table A.1, Supplementary data).

### Reasons for revision

A total of 104 (1.4%) DM cups had been revised since the start (January 1, 2007) until the end of the follow-up period (January 1, 2021). In addition, 130 (1.7%) head/liners had been revised. The most common reasons for DM cup revision were infection (25%) and acetabular loosening (22%). The most frequently registered reason for head/liner revision was infection (70%). In total, 48 of 104 (46%) of the revised DM cups and 18 of 130 (14%) of the revised liners were cup or liner related (wear, dislocation, or acetabular loosening) (Table 2).

**Table 2:** Reasons for DM cup or head/liner revision for primary THA with a DM cup in the period 2007-2020 in the Netherlands (n = 7603 THAs of which 234 (3.1%) were revised) categorized into cup related and other revisions.

Reason for DM cup revision	Cup revision, n (%)	Head/Liner revision,^a^ n (%)	Total cup or head/liner revision, n (%)
7603 (100%) DM cups	104 (1.4%)	130 (1.7%)	234 (3.1%)
Cup- or liner-related revisions			
Wear (liner)	2	1	3
Dislocation	13	15	28
Acetabular loosening	22	0	22
Dislocation + acetabular loosening	6	0	6
Dislocation + wear	3	2	5
Acetabular loosening + dislocation + wear	2	0	2
Total cup- or liner-related revisions	48 (0.6%)	18 (0.2%)	66 (0.9%)
Other revisions			
Infection	26	92	118
Femoral loosening	3	0	3
Loosening + infection	9	0	9
Periprosthetic fracture	6	1	7
Other	12	19	31
Total other revision	56 (0.7%)	112 (1.5%)	168 (2.2%)

DM, dual mobility; THA, total hip arthroplasty.

a Head/liner revision only (no acetabular cup revision).

### Risk of revision

**Figure 3:**
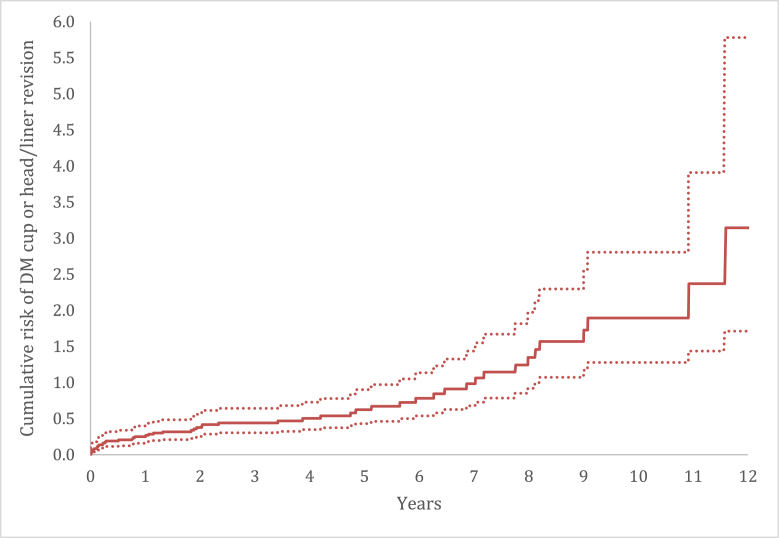
Crude cumulative incidence of DM cup or head/liner revision (for dislocation, wear or acetabular loosening) of primary THAs with a DM cup performed in 2007-2020 in the Netherlands.

The crude cumulative incidence of DM cup or head/liner revision for dislocation, liner wear, and acetabular loosening after 5 and 10 years was 0.5% (CI 0.4-0.8) and 1.9% (CI 1.3-2.8), respectively, (Fig. 3).

### Femoral neck design

**Figure A.1:**
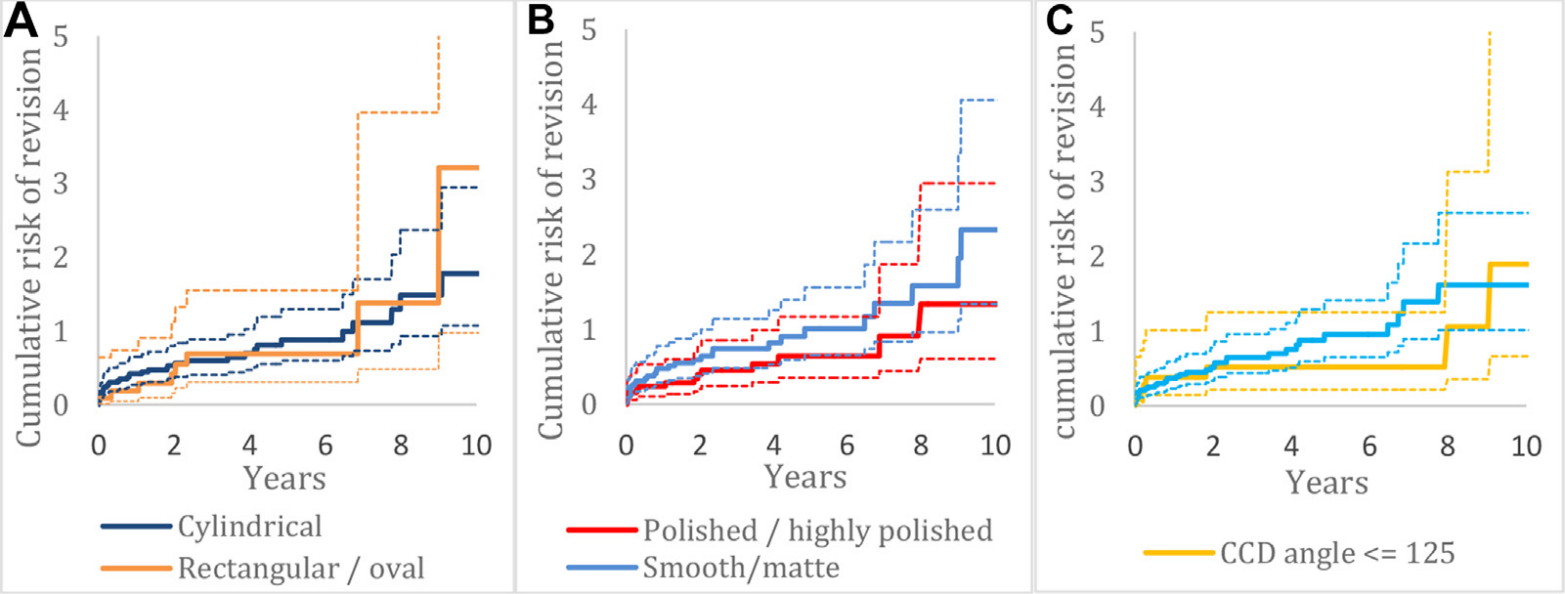
Crude cumulative incidence of DM cup or head/liner revision (for dislocation, wear, or acetabular loosening) of primary THAs performed in 2007-2020 in the Netherlands according to (a) femoral neck geometry, (b) neck surface roughness, and (c) CCD angle.

After selection of the most frequently used (n > 150) femoral components, we included 6170 THAs for analysis. Among the different alloys used for femoral neck implants, 3 types were compared: stainless steel, titanium, and cobalt-chrome (Table 3). The adjusted HRs for revision were comparable between the different alloys with cobalt chrome as a reference (respectively, stainless steel HR 0.4 [CI 0.1-1.8] and titanium HR 1.8 [CI 0.9-3.6]). Two different neck geometries were examined: rectangular/oval and cylindrical (Fig. A.1a, Appendix). The adjusted revision rate for THAs with a rectangular/oval femoral neck design (HR 0.8 [CI 0.4-1.9]) was comparable with THAs with a cylindrical neck design. Different CCD angles were included among the various femoral stems within the data set. The CCD angle was categorized into 2 groups: ≤125 and >125. We found a comparable risk of revision for prostheses with a CCD angle ≤125 (HR 0.6 [CI 0.3-1.6]) compared with prostheses with a CCD angle >125 (Fig. A.1b, Appendix). For neck surface roughness, implants with a polished/highly polished neck surface were compared with THAs with a smooth/matte neck surface (Fig. A.1c, Supplementary data). The adjusted risk of revision for polished/highly polished necks (HR 0.5 [CI 0.3-1.0]) did not differ from femoral necks with a matte surface. Finally, we found that neither bearing material nor femoral head size was associated with an increased risk of revision due to dislocation, wear, or acetabular loosening of the DM cup or head/liner (Tables 1 and 3).

**Table 3:** Multivariable survival analysis (relative risks of cup or head/liner revision due to dislocation, wear and loosening according to prosthesis characteristics).

Femoral neck characteristics	Number of procedures, N = 6170^b^	Revisions, n (%)^b^	Crude hazard ratio (CI)	Adjusted hazard ratio (CI)^c^
Femoral neck characteristics				
Aloy used				
Stainless steel	771	2 (0.3)	0.4 (0.1-1.8)	0.4 (0.1-1.8)
Titanium	1550	23 (1.5)	2.2 (1.2-4.1)^a^	1.8 (0.9-3.6)
Cobalt chrome	3849	20 (0.5)	1.0	1.0
Femoral neck geometry				
Rectangular	1117	8 (0.7)	1.0 (0.5-2.2)	0.8 (0.4-1.9)
Cylindrical	5053	37 (0.7)	1.0	1.0
CCD angle				
≤125	1103	7 (0.6)	0.7 (0.3-1.6)	0.6 (0.3-1.6)
>125	4886	36 (0.7)	1.0	1.0
Neck surface roughness				
Polished/highly polished neck	2569	11 (0.4)	0.5 (0.3-0.9)^a^	0.5 (0.3-1.0)
Smooth/Matte neck surface	3601	34 (0.9)	1.0	1.0
Femoral head characteristics				
Femoral head size				
22 mm	515	2 (0.4)	0.5 (0.1-2.0)	0.5 (0.1-2.1)
28 mm	5519	41 (0.7)	1.0	1.0
Bearing material				
PE Standard	2953	33 (1.1)	1.3 (0.5-3.8)	1.2 (0.4-3.5)
PE Cross-linked + Antioxidant	2381	6 (0.3)	0.4 (0.1-1.4)	0.4 (0.1-1.5)
PE Cross-linked	612	4 (0.7)	1.0	1.0

ASA, American Society of Anesthesiology score; CI, confidence interval; PE, polyethylene.

a *P* < .05.

b Numbers do not add up due to missing values.

c Adjusted for age, sex, ASA-score and diagnosis.

## Discussion

In this observational study with data from the Dutch Arthroplasty Register, we examined the reasons for revision of DM cups and head/liners (n = 7603). Specifically, we examined the risk of cup-related revision (dislocation, wear, and acetabular loosening). Furthermore, we explored whether certain femoral neck characteristics in combination with DM cup are associated with a higher acetabular cup or head/liner revision rate for dislocation, wear, and acetabular loosening. The most common reason for DM cup and head/liner revision was infection. The DM cup or head/liner revision rate for cup-related revisions (wear, dislocation, and acetabular loosening) at 10 years was low (1.9%). Femoral neck characteristics did not influence the risk of DM cup or head/liner revision. These findings suggest that in general, the most frequently used femoral stems are suitable to be combined with DM cups.

### Reasons of revision

The first-generation DM cups were associated with higher aseptic loosening and IPD rate, which resulted from PE wear, suboptimal fixation, and surface coating of the acetabular cup [12,[32], [33], [34]]. The incidence of IPD was reduced by modifications to the DM implants including smooth, polished, and narrow necks, the use of HXPLE liners, and modifications to liner design [11,35]. The findings of our study strongly indicate that the risk of revision specifically for wear, dislocation, and acetabular loosening in DM cups and head/liners is low. Most DM cup studies have been focusing on the overall implant survival and failure modes of this unique design compared to unipolar cups, which makes it challenging to directly compare our results with existing literature. A population-based prospective cohort study using the Nordic Arthroplasty Register Association database, including 2227 primary procedures, reported a cumulative revision rate of 4.1% for DM cup THPs after 9 years [1]. The authors reported that, despite being one of the most frequently registered reasons for revision (16%), revision rates for acetabular loosening were low (16/2277 [0.7%]). Moreover, the study reported low revision rates for dislocation (2/2277 [0.1%]). However, it is important to note that the study encompassed a broad range or revision procedures without focusing on specific components such as DM cups or liners. Unlike the previously mentioned study, a population-based cohort study by Bloemheuvel et al, specifically examined the rates and reasons for DM cup revisions [5]. The authors analyzed 3038 primary DM cup THAs from the LROI between 2007 and 2016 and reported an overall 5-year revision rate of 1.5% (95% CI: 1.0-2.3), with acetabular loosening being the most frequently registered reason for revision (0.5%). The number of revisions due to dislocation (0.2%) and liner wear (0%) was low. In our study, we found that wear, dislocation, and acetabular loosening were responsible for 46% of the registered revisions of DM cups. These specific revisions only accounted for 0.6% of all primary DM cups. These results suggest that while wear, dislocation, and acetabular loosening are factors contributing to DM cup revisions, the overall risk of revision specifically for these issues is low.

We hypothesized that prosthetic impingement and liner wear due to repeated friction and loading contribute to acetabular loosening or alternatively to dislodgment of the liner. Unfortunately, specific reasons such as prosthetic impingement were not explicitly recorded in the LROI. Scot et al (2018) examined the occurrence of implant-related impingement after THA using a DM cup construct [36]. The study revealed that DM cup liners had an impingement rate of 22%, which was significantly lower than the impingement rate observed in primary fixed-bearing THA liners in a previous study they performed [37]. Surprisingly, despite the expectation of increased contact between DM cup liners and the femoral neck, the study found no evidence of increased damage.

### Femoral neck design

To date, limited research has been performed on femoral neck and taper design in relation to clinical outcome of THA using DM cups. Although the risk of revision for wear, dislocation, and loosening is relatively low, it is important to consider which factors may be associated with the failure of DM implants. Wegrzyn et al (2022) investigated the impact of geometry, surface finishing roughness on PE damage, and wear in DM cups [38]. The study reported that femoral neck characteristics did not significantly affect PE damage and wear to the liner, except in cases of restricted motion where fixed femoral neck impingement occurred. In such scenarios, quadrangular femoral necks resulted in higher PE damage and wear than cylindrical femoral necks, particularly when combined with rough surface finishing. Conversely, surface finishing roughness did not impact PE damage and wear with the cylindrical geometry regardless of the impingement condition. Another study from Di Laura (2017) involving 70 DM cups examined the impact of 2 femoral stem designs from the same manufacturer on PE impingement [39]. The authors found that femoral stems with a smooth femoral neck design (Rejuvenate, Stryker Orthopaedics, Mahwah, NJ) had a lower incidence of liner rim deformations caused by impingement from the femoral neck than a sharper femoral neck geometry and rougher surface finishing (ABGII, Stryker). A third study by Phillipot (2013) followed 1850 patients who received THA using a DM cup between 1985 and 1998 [36]. The study involved 2 types of femoral stems: stainless steel PF (Serf, Decines, France) with a large neck diameter and titanium PRO (Serf) with a smaller neck diameter (16 mm and 13 mm, respectively). The authors reported no difference in IPD rates between the 2 stems. The authors hypothesized that the absence of significant differences could be attributed to various dissimilarities in prosthesis characteristics. They hypothesized that the PF stem's large neck diameter and the PRO stem's unpolished titanium surface both increased the risk of IPD [36]. Together, these studies highlight the potential impact of femoral neck characteristics on wear and the associated complications that may arise. Despite a relatively high number of THAs in our study, we did not find clinical differences to support these theories.

### Limitations

Our findings should be interpreted in the context of several limitations. First, a number of femoral neck characteristics (eg, femoral neck diameter) could not be determined since characteristics were not available within the LROI implant library, manufacturer implant brochures, or other publicly accessible sources. Some large orthopedic manufacturers were reluctant to provide these details on our request. Moreover, we acknowledge the limitation of not evaluating crucial factors such as cup position, head-neck ratio, and cup size. For example, an excessively flat cup or one with excessive anteversion or retroversion can increase the likelihood of neck impingement, irrespective of femoral neck taper design. Moreover, an inadequate head-neck ratio can result in impingement and cause friction, wear, and dislocation of the DM implant. These characteristics could potentially impact the overall conclusions drawn from the study. However, the absence of such data in our data set prevented their evaluation in our study. Third, this study may not establish a direct cause-and-effect relationship between femoral neck geometry and the outcomes of interest, since an observational study design was used. The median age of our patient population was 73 years, and it is important to acknowledge that older patients may experience different outcomes compared to younger cohorts. This age-related bias could potentially affect the generalizability of our results; hence, future research should consider including a broader age range. Finally, the occurrence of IPD cannot be exactly determined. In the current LROI registration form, IPD cannot be selected as an option for reasons such as revision and is therefore registered as “dislocation.” Hence, we assume that when a DM cup is revised for dislocation, this could be the result of IPD. Lastly, postoperative dislocations treated with closed reduction were not registered in the LROI since no component change was carried out.

### Implications for clinical practice

In vitro studies suggest a possible relationship between femoral neck design and PE wear, potentially increasing the risk of revision. However, we did not find clinical evidence for these theories. Therefore, this study emphasizes that the most frequently used femoral stems in the Netherlands in combination with a DM cup can be safely combined. Future clinical studies are needed to develop a deeper understanding of the relationship between femoral neck characteristics and DM cup revision.

## Conclusions

The risk of DM cup or head/liner revision for wear, dislocation, and acetabular loosening is low. We found no association between femoral neck design and the risk of revision for wear, dislocation, or acetabular loosening. Taken together, these findings do not support strong recommendations in favor of specific femoral neck design features in combination with DM constructs in primary THA.

## Conflicts of interest

The authors declare there are no conflicts of interest.

For full disclosure statements refer to https://doi.org/10.1016/j.artd.2023.101281.

## Authors’ contributions

Liza N van Steenbergen: Conceptualization, Methodology, Supervision, Writing – original draft, Writing – review & editing. P Koen Bos: Conceptualization, Data curation, Investigation, Methodology, Supervision, Visualization, Writing – original draft, Writing – review & editing. Rinne M Peters: Conceptualization, Formal analysis, Investigation, Methodology, Supervision, Writing – original draft, Writing – review & editing. David Visser: Investigation, Methodology, Writing – original draft, Writing – review & editing. Bart-Jan van Dooren: Conceptualization, Data curation, Formal analysis, Investigation, Methodology, Resources, Validation, Visualization, Writing – original draft, Writing – review & editing. Wierd P Zijlstra: Conceptualization, Data curation, Investigation, Methodology, Supervision, Visualization, Writing – original draft, Writing – review & editing.

## Figures and Tables

**Table A.1:** Descriptive statistics for primary DM cup THAs in the Netherlands between 2007 and 2020 (n = 7603).

Patient and procedure characteristics	DM cup THAs total (n = 7603)^b^
Factor	N (%)^b^
Sex^b^	
Male	2687 (35)
Female	4909 (65)
Age (years), median (SD)	73 (12)
Previous surgery of affected joint^b^	
Yes	1517 (20)
No	5940 (80)
ASA score^b^	
I	579 (8)
II	4093 (54)
III-IV	2877 (38)
Diagnosis^b^	
Osteoarthritis	3955 (52)
Non-osteoarthritis	3593 (48)
BMI (kg/m^2^)^a^^,^^b^	
<18.5	200 (3)
18.5-25	2610 (41)
25-30	2234 (36)
30-40	1129 (18)
>40	140 (2)
Fixation^b^	
Cemented femoral stem + cup	5036 (67)
Cementless femoral stem + cup	1132 (15)
Reversed hybrid (cemented cup)	1043 (14)
Hybrid (cementless cup)	289 (4)
Head size^b^	
22 mm	642 (9)
28 mm	6677 (91)
Articulation^b^	
Ceramic on PE	3072 (43)
Metal on PE	3835 (53)
Zirconium on PE	287 (4)
Surgical approach^b^	
Straight lateral	391 (5)
Posterolateral	6707 (89)
Anterolateral	72 (1)
Anterior	312 (4)
Other	82 (1)

ASA score, American Society of Anesthesiology score; BMI, body mass index.

a Available since 2014.

b Numbers do not add up to total due to unknown or missing values.

## Appendix A Supplementary data

Conflict of Interest Statement for Visser

Conflict of Interest Statement for van Dooren

Conflict of Interest Statement for Bos

Conflict of Interest Statement for Peters

Conflict of Interest Statement for van Steenbergen

Conflict of Interest Statement for Zijlstra
