# Supplementary material for: Femoral Neck Design Does Not Impact Revision Risk After Primary Total Hip Arthroplasty Using a Dual Mobility Cup
Source: Arthroplast Today. 2024 Jan 13;25:101281. doi: 10.1016/j.artd.2023.101281 (PMC10826135; doi:10.1016/j.artd.2023.101281)
Supplement: Conflict of Interest Statement for Zijlstra [file mmc6.pdf]

# INDIVIDUAL CONFLICT OF INTEREST STATEMENT

## *American Association of Hip and Knee Surgeons*

(Adopted from the American Academy of Orthopaedic Surgeons disclosure statement)

The following form **must be filled out completely and submitted by each author (example, 6 authors, 6 forms). All items require a response. If there is no relevant disclosure for a given item, enter "*None*."**

**Manuscript Title:** Femoral neck design does not impact revision risk after primary total hip arthroplasty (THA) using a dual mobility cup: Results from 7,603 primary THAs in the Dutch Arthroplasty Register (LROI).

1. Royalties from a company or supplier (The following conflicts were disclosed)
**None**

2. Speakers bureau/paid presentations for a company or supplier (The following conflicts were disclosed)
**None**

3A. Paid employee for a company or supplier (The following conflicts were disclosed)
**None**

3B. Paid consultant for a company or supplier (The following conflicts were disclosed)
**None**

3C. Unpaid consultants for a company or supplier (The following conflicts were disclosed)
**None**

4. Stock or stock options in a company or supplier (The following conflicts were disclosed)
**None**

5. Research support from a company or supplier as a Principal Investigator (The following conflicts were disclosed)
**Dr. Zijlstra reports financial support provided to the hospital (Medical Center Leeuwarden) by Smith & Nephew. This was not related to the study.**

6. Other financial or material support from a company or supplier (The following conflicts were disclosed)
**None**

7. Royalties, financial or material support from publishers (The following conflicts were disclosed)
**None**

8. Medical/Orthopaedic publications editorial/governing board (The following conflicts were disclosed)
**None**

9. Board member/committee appointments for a society (The following conflicts were disclosed)
**None**

**Each author must sign AND print or type his/her name, date and submit a separate form**

In addition, one BLINDED Conflict of Interest form (no author names used) should be submitted per manuscript with all author disclosures.

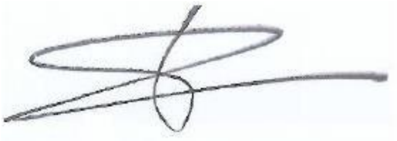

| W.P. Zijlstra | | Sept 6th, 2023 |
| --- | --- | --- |
| Author Name (Print or Type) | Author Signature | Date |
